# Should emergency physicians be included in the atrial fibrillation heart team?

**DOI:** 10.3389/fcvm.2026.1801655

**Published:** 2026-05-19

**Authors:** Jerica Zaloznik Djordjevic, Katsiaryna Yermak, Anze Djordjevic

**Affiliations:** 1 Department of Emergency Medicine, University Medical Centre Maribor, Maribor, Slovenia; 2 Department of Internal Medicine and Cardiology, Oberhavel Kliniken GmbH, Klinik Hennigsdorf, Hennigsdorf, Germany; 3 Department of Cardiac Surgery, University Medical Centre Maribor, Maribor, Slovenia

**Keywords:** anticoagulation, atrial fibrillation, care pathways, emergency medicine, integrated care

## Abstract

Atrial fibrillation is an expanding global health challenge associated with increasing prevalence, substantial morbidity, and growing healthcare costs. Most symptomatic AF encounters begin in the emergency department, yet emergency physicians are frequently excluded from formal AF “heart-team” structures. Contemporary models of AF care emphasize multidisciplinary coordination, recognizing the complex interplay of electrophysiological, structural, inflammatory, and systemic factors that influence AF onset, persistence, and long-term outcomes. Evidence demonstrates that ED-based management—particularly a decision about an early rate or rhythm control, anticoagulation decisions, and structured discharge pathways—significantly affects downstream care quality and patient safety. This editorial argues that emergency physicians are indispensable members of AF heart teams. Their inclusion enhances the integration of acute and longitudinal management, promotes guideline adherence, and addresses persistent gaps in early decision-making that shape the course of AF care.

## Introduction

Atrial fibrillation (AF) has become one of the most prevalent and costly cardiovascular disorders worldwide (1). Its incidence continues to rise, with demographic aging, improved survival from cardiovascular disease, and increasing prevalence of lifestyle-related risk factors all contributing to a sharply expanding patient population (2, 3). In parallel, AF imposes a substantial financial burden due to AF-related complications, hospitalizations, stroke prevention strategies, rhythm-control interventions, and long-term monitoring (4, 5).

The growing complexity of AF management has prompted a shift toward integrated, multidisciplinary approaches. Cardiologists, electrophysiologists, cardiac surgeons, community nurses, general practitioners and pharmacologists all contribute to the contemporary AF care pathway. Emergency physicians are providing the earliest care of atrial fibrillation, yet they remain systematically excluded from the heart team, despite their pivotal role in the initial diagnostic and therapeutic phase of AF management. This omission limits the coherence of care across transitions and overlooks a specialty that profoundly influences the trajectory of AF management.

### AF as a complex, multisystem disorder

A major reason to expand AF heart-team membership lies in AF's multifactorial pathophysiology. Research across surgical and medical populations demonstrates that atrial remodelling is driven not only by electrophysiological and structural changes but also by oxidative stress, mitochondrial dysfunction, and inflammation (6–8). Studies examining postoperative AF, for example, show how oxidative injury and metabolic stress amplify vulnerability to atrial arrhythmias, emphasizing that AF arises from dynamic and systemic biological processes rather than isolated electrical triggers (9).

Interventional, surgical and combined hybrid treatment options for atrial fibrillation are evolving rapidly, supported by technological advances that enable safer procedures and more durable lesion formation (10). However, reducing morbidity and mortality and aiming for sustained rhythm control still depend critically on the systematic treatment of modifiable risk factors (11, 12). To ensure optimal outcomes, all therapeutic strategies must be well coordinated and effectively communicated among members of the heart team. Moreover, patient education and the inclusion of patient preferences in the formulation of treatment goals significantly enhance treatment adherence (13, 14).

Integrating emergency physicians into this continuum ensures that early clinical assessments—such as recognizing precipitating factors, identifying high-risk phenotypes, and initiating early protective therapies—fit seamlessly into long-term management plans.

### Emergency physicians at the front line of key decisions

The ED remains the first point of medical contact for a large proportion of patients with symptomatic or newly diagnosed AF. During this encounter, emergency physicians must rapidly assess hemodynamic stability, identify reversible triggers, determine stroke and bleeding risks, assess the patient's symptoms and choose between rate and rhythm control strategies. They also decide whether to anticoagulate, perform cardioversion, admit the patient, or arrange expedited outpatient follow-up (15, 16).

These decisions carry substantial downstream consequences. Initiation of appropriate anticoagulation is one of the most critical early steps in AF management, yet it remains inconsistently implemented in ED discharges across many health systems (17, 18). Early rhythm-control strategies, when used selectively, can reduce symptom burden and may influence longer-term outcomes. Moreover, the ED disposition decision—whether a patient is safely discharged with structured follow-up or admitted for inpatient evaluation—shapes resource utilization and care continuity (19–21).

In addition, emergency physicians play a crucial role in recognizing acute complications of atrial fibrillation therapies—such as bleeding events related to oral anticoagulation or adverse reactions to antiarrhythmic drugs—allowing them to modify treatment strategies, document therapy failures, and serve as key coordinators within the multidisciplinary care pathway.

A clear distinction should be made between first-diagnosed and recurrent atrial fibrillation, as the scope of emergency department decision-making differs substantially between these presentations. In first-diagnosed AF, emergency physicians manage greater diagnostic uncertainty and a broader clinical mandate, including confirmation of the diagnosis, identification of underlying and reversible causes, risk stratification, and initiation of guideline-directed therapy. Early decisions regarding anticoagulation and rate or rhythm control frequently determine downstream management and long-term outcomes. In recurrent AF, the clinical focus shifts toward assessment of hemodynamic stability, identification of precipitating factors or complications, symptom control, and reassessment of established treatment strategies. Acknowledging these distinct scenarios further supports the integration of emergency physicians into the atrial fibrillation heart team, as their role is critical across both initial and recurrent phases of care.

### The alignment between ED practice and integrated guideline frameworks

Modern AF guidelines propose a holistic, structured care model emphasizing stroke prevention, symptom management, and modification of cardiovascular risk factors (6). This “ABC” framework is designed to unify clinical practice across healthcare environments. However, effective implementation depends on consistent application beginning with the first clinical encounter.

If emergency physicians are not part of the heart team, opportunities to initiate the ABC framework are often missed. For example, decisions regarding anticoagulation may be delayed or deferred; early rhythm-control considerations may not align with electrophysiology strategy; and cardiovascular risk assessment may be postponed until outpatient review. These delays can lead to avoidable variation, fragmented transitions, and reduced adherence to guideline-directed therapy.

Emergency physicians bring valuable insight into how guidelines can be translated into efficient, safe, and actionable protocols within the fast-paced ED environment. Their expertise helps ensure that standardized pathways are realistic and that early-care decisions align with long-term goals.

### Benefits of including emergency physicians in the AF heart team

Integrating emergency physicians into the AF heart team offers several advantages. Their participation supports the development of coordinated clinical pathways - and strengthen adherence to evidence-based practice, particularly regarding anticoagulation initiation and safe cardioversion approaches. Collaboration with ED clinicians also facilitates the creation of effective transitions-of-care mechanisms, such as rapid-access to AF specialists for symptomatic patients, advise primary physicians about treatment options and follow-ups, which improve continuity and reduce unnecessary hospitalizations.

From a system perspective, emergency physicians contribute a practical understanding of workflow, triage, and real-world constraints. Their frontline experience positions them ideally to participate in quality-improvement initiatives and to monitor key clinical performance metrics, including ED revisits, safe discharge rates, and the timeliness of outpatient evaluation. This comprehensive perspective enhances the heart team's ability to design strategies that are both clinically robust and operationally feasible.

## Conclusion

Atrial fibrillation is a complex, multisystem disorder requiring coordinated decision-making across specialties and care settings. As research continues to reveal new dimensions of AF mechanisms and stroke-prevention strategies, the importance of a fully integrated heart team becomes even clearer. Emergency physicians, as the clinicians who guide the earliest and most time-sensitive decisions, are essential members of this team. Their inclusion enhances continuity, improves guideline adherence, and ensures that AF care reflects the entire patient journey—from the first symptomatic episode to long-term management. In the modern era of AF care, a heart team without emergency physicians is incomplete.

## References

[1] Sandhu R Qureshi H Dover D Hawkins MN Kaul P. The current and future total health care costs of atrial fibrillation. Eur Heart J. (2023) 44:ehad655.438. 10.1093/eurheartj/ehad655.438

[2] Go AS Hylek EM Phillips KA Chang Y Henault LE Selby JV Prevalence of diagnosed atrial fibrillation in adults: national implications for rhythm management and stroke prevention: the AnTicoagulation and risk factors in atrial fibrillation (ATRIA) study. JAMA. (2001) 285(18):2370–5. 10.1001/jama.285.18.2370 11343485

[3] Hindricks G Potpara T Dagres N Arbelo E Bax JJ Blomström-Lundqvist C 2020 ESC guidelines for the diagnosis and management of atrial fibrillation developed in collaboration with the European association for cardio-thoracic surgery (EACTS): the task force for the diagnosis and management of atrial fibrillation of the European Society of Cardiology (ESC) developed with the special contribution of the European heart rhythm association (EHRA) of the ESC. Eur Heart J. (2021) 42(5):373–498. 10.1093/eurheartj/ehaa612 32860505

[4] Delaney JA Yin X Fontes JD Wallace ER Skinner A Wang N Hospital and clinical care costs associated with atrial fibrillation for medicare beneficiaries in the cardiovascular health study and the framingham heart study. SAGE Open Med. (2018) 6:2050312118759444. 10.1177/2050312118759444 29511541 PMC5826000

[5] Walli-Attaei M Luengo-Fernandez R Gray A Torbica A Maggioni AP Bairami F Variations in the costs of atrial fibrillation a longitudinal study across 27 European and central Asian countries. Eur Heart J. (2023) 44:ehad655.3032. 10.1093/eurheartj/ehad655.3032

[6] Tzeis S Gerstenfeld EP Kalman J Saad EB Sepehri Shamloo A Andrade JG 2024 European heart rhythm association/heart rhythm society/Asia Pacific heart rhythm society/Latin American heart rhythm society expert consensus statement on catheter and surgical ablation of atrial fibrillation. Europace. (2024) 26(4):euae043. 10.1093/europace/euae043 38587017 PMC11000153

[7] Vercek G Jug B Novakovic M Antonic M Djordjevic A Ksela J Conventional and novel inflammatory biomarkers in chronic heart failure patients with atrial fibrillation. Medicina (Kaunas). (2024) 60(8):1238. 10.3390/medicina60081238 39202519 PMC11356261

[8] Brundel BJJM Ai X True Hills M Kuipers MF Lip GYH de Groot NMS. Atrial fibrillation. Nat Rev Dis Primers. (2022) 8(1):21. 10.1038/s41572-022-00347-9 35393446

[9] Intihar U Krasniqi A Djordjevic A Zmazek J Avdagic H Ksela J Oxidative stress in postoperative atrial fibrillation: does malondialdehyde hold predictive value? Medicina (Kaunas). (2025) 61(5):778. 10.3390/medicina61050778 40428736 PMC12113208

[10] Wong CX Buch EF Beygui R Lee RJ. Hybrid endo-epicardial therapies for advanced atrial fibrillation. J Clin Med. (2024) 13(3):679. 10.3390/jcm13030679 38337373 PMC10856493

[11] Pathak RK Middeldorp ME Lau DH Mehta AB Mahajan R Twomey D Aggressive risk factor reduction study for atrial fibrillation and implications for the outcome of ablation: the ARREST-AF cohort study. J Am Coll Cardiol. (2014) 64(21):2222–31. 10.1016/j.jacc.2014.09.028 25456757

[12] Pathak RK Middeldorp ME Meredith M Mehta AB Mahajan R Wong CX Long-Term effect of goal-directed weight management in an atrial fibrillation cohort: a long-term follow-up study (LEGACY). J Am Coll Cardiol. (2015) 65(20):2159–69. 10.1016/j.jacc.2015.03.002 25792361

[13] Vinereanu D Lopes RD Bahit MC Xavier D Jiang J Al-Khalidi HR A multifaceted intervention to improve treatment with oral anticoagulants in atrial fibrillation (IMPACT-AF): an international, cluster-randomised trial. Lancet. (2017) 390(10104):1737–46. 10.1016/S0140-6736(17)32165-7 28859942

[14] Desteghe L Delesie M Knaepen L Önder R Verbeeck J Dendale P Effect of targeted education of patients with atrial fibrillation on unplanned cardiovascular outcomes: results of the multicentre randomized AF-EduCare trial. Europace. (2024) 27(1):euae211. 10.1093/europace/euae211 39832209 PMC11745127

[15] Caldarola P De Iaco F Pugliese FR De Luca L Fabbri A Riccio C ANMCO-SIMEU consensus document: appropriate management of atrial fibrillation in the emergency department. Eur Heart J Suppl. (2023) 25(Suppl D):D255–77. 10.1093/eurheartjsupp/suad110 37213798 PMC10194824

[16] Joglar JA Chung MK Armbruster AL Benjamin EJ Chyou JY Cronin EM 2023 ACC/AHA/ACCP/HRS guideline for the diagnosis and management of atrial fibrillation: a report of the American College of Cardiology/American Heart Association joint committee on clinical practice guidelines. Circulation. (2024) 149(1):e1–e156. 10.1161/CIR.0000000000001193 38033089 PMC11095842

[17] Rezazadeh S Chew DS Miller RJH Klassen S Pournazari P Bennett G Effects of a reminder to initiate oral anticoagulation in patients with atrial fibrillation/atrial flutter discharged from the emergency department: rEMINDER study. CJEM. (2018) 20(6):841–9. 10.1017/cem.2018.415 30295590

[18] Habeeb E Papadopoulos T Lewin AR Knowles D. Assessment of anticoagulant initiation in patients with new-onset atrial fibrillation during emergency department visit-point-by-point response. Clin Appl Thromb Hemost. (2023) 29:10760296231172493. 10.1177/10760296231172493 37138471 PMC10161192

[19] Ptaszek LM Baugh CW Lubitz SA Ruskin JN Ha G Forsch M Impact of a multidisciplinary treatment pathway for atrial fibrillation in the emergency department on hospital admissions and length of stay: results of a multi-center study. J Am Heart Assoc. (2019) 8(18):e012656. 10.1161/JAHA.119.012656 31510841 PMC6818017

[20] Barbic D DeWitt C Harris D Stenstrom R Grafstein E Wu C Implementation of an emergency department atrial fibrillation and flutter pathway improves rates of appropriate anticoagulation, reduces length of stay and thirty-day revisit rates for congestive heart failure. CJEM. (2018) 20(3):392–400. 10.1017/cem.2017.418 29117873

[21] Addy K Joyce LR Al-Busaidi IS Pickering JW Troughton R Than M Implementation of an integrated emergency department acute atrial fibrillation pathway safely reduces cardioversions and hospitalisations: a comparative pre-post study. Emerg Med Australas. (2023) 35(5):828–33. 37169715 10.1111/1742-6723.14240

